# Adaptive Attentional Regulation to Emotional Faces in Subclinical Depression

**DOI:** 10.3390/bs16050657

**Published:** 2026-04-26

**Authors:** Chaoyang Li, Jinhong Ding

**Affiliations:** School of Psychology, Capital Normal University, Beijing 100048, China; 2213501009@cnu.edu.cn

**Keywords:** visual search, subclinical depression, attentional bias, emotion regulation, eye-tracking

## Abstract

Cognitive models of depression posit a core role for attentional biases, though empirical evidence remains inconsistent, likely due to variations in task demands. This study utilized eye-tracking to assess attentional patterns in individuals with depressive symptoms during a goal-directed visual search task, specifically dissociating early orienting and late disengagement. Seventy-seven participants, classified into high (HD) and low (LD) depressive-symptom groups based on PHQ-9 scores, completed a “face-in-the-crowd” (FITC) task. The set size (4, 8, or 12 faces) was varied to examine the role of perceptual load. The task involved searching for a single emotional target among neutral distractors (assessing early orienting) and searching for a single neutral target among emotional distractors (assessing late disengagement). Contrary to the negativity-bias hypothesis, the HD group demonstrated what might be interpreted as adaptive attentional regulation. During early orienting (8-face condition), the HD group showed reduced total dwell time on happy targets, suggesting accelerated identification. An attentional bias index (sad minus happy dwell time) correlated positively with depression severity. During late disengagement (8-face condition), the HD group exhibited shorter target fixation latency specifically with sad distractors, indicating facilitated disengagement from negative information. The corresponding bias index correlated negatively with depression levels. Under explicit goal-directed demands, individuals with high depressive symptoms displayed facilitated processing of happy faces and accelerated disengagement from sad faces, rather than an enhanced negativity bias. This pattern tentatively suggests a possible adaptive attentional regulatory mechanism in early depression, although the findings were limited to the 8-face condition and no significant group differences emerged at set sizes 4 or 12. Replication is required before firm conclusions can be drawn. The result underscores the critical influence of task demands and highlights the value of early identification and targeted intervention.

## 1. Introduction

Major depressive disorder (MDD) is a prevalent global mental health condition, affecting approximately 180 million people and imposing a substantial burden on both individuals and healthcare systems (Marx et al., 2023; Herrman et al., 2022; Kupferberg et al., 2016). Core features include persistent low mood, anhedonia, and recurrent suicidal ideation. Cognitive models of depression posit that these symptoms arise and persist due to dysregulated attention toward emotional information (Beck & Haigh, 2014; Gotlib & Joormann, 2010; LeMoult & Gotlib, 2019). This dysregulation is characterized by two key attentional biases: first, a heightened maintenance of attention on negative over neutral or positive information (De Raedt & Koster, 2010; Peckham et al., 2010), and second, a lack of the protective bias toward positive information seen in healthy individuals (Duque & Vázquez, 2015; Isaac et al., 2014; Winer & Salem, 2016). Consequently, individuals with MDD may be unable to leverage positive information for effective emotion regulation, a process that likely contributes to the exacerbation of depressive symptoms (Moretta et al., 2021).

Research on attentional bias in affective disorders has traditionally relied on reaction time (RT) tasks, such as the dot probe, to infer attention allocation (Peckham et al., 2010). However, these RT-based paradigms have significant limitations. They often demonstrate insufficient reliability (Chapman et al., 2019; Lazarov et al., 2021; Price et al., 2015; Weierich et al., 2008), and RT is an indirect measure of emotional response. The temporal separation between stimulus presentation and behavioral response can introduce confounding factors (Hertz-Palmor et al., 2024; Lazarov et al., 2021), potentially leading to inconsistent and difficult-to-replicate findings (Klawohn et al., 2020; Mogg & Bradley, 2005). In contrast, eye-tracking technology directly measures attentional allocation and its temporal dynamics, thereby overcoming the key shortcomings of RT measures (Armstrong & Olatunji, 2012; Lazarov et al., 2021).

Eye-tracking studies of attentional bias primarily utilize free-viewing and visual search paradigms. In free-viewing tasks, participants observe a series of stimuli (e.g., emotional faces) without specific instructions, thereby revealing spontaneous, naturalistic attention patterns (Lazarov et al., 2018). In contrast, visual search tasks require participants to identify a target among distractors, such as a negative face among neutral ones (Bodenschatz et al., 2021; Suslow et al., 2021). Here, an attentional bias facilitates faster target detection, indicating reduced interference from distractors. The free-viewing paradigm is more commonly used, and findings from it suggest that the attentional bias in depression may not lie in initial orienting, but rather in prolonged maintenance on negative information and diminished maintenance on positive information (Suslow et al., 2020).

Bodenschatz et al. (2021) distinguished between the attentional biases captured by different paradigms. Free-viewing tasks, which are passive and lack external demands, reveal spontaneous, stimulus-driven bias but are less effective for assessing goal-directed cognitive processes. In contrast, visual search tasks, with their explicit targets, require active processing and thus probe goal-directed attention. However, visual search studies have predominantly used reaction time measures with inconsistent results (Suslow et al., 2004; Wisco et al., 2012). To address this, Bodenschatz et al. (2021) employed an eye-tracking face-in-the-crowd task, where participants identified a discrepant emotional face among eight. They found no significant differences between depressed and non-depressed individuals in either reaction times or eye movements. The authors proposed that explicit task demands may override a latent attentional bias in depressed individuals, allowing them to perform similarly to controls. This suggests that proactive, goal-directed attentional bias in depression may differ fundamentally from passive, stimulus-driven bias.

This discrepancy may arise from variations in either task requirements or the experimental materials employed. While attentional bias toward angry faces has been identified as a potential risk factor for depression (Woody et al., 2016), evidence remains inconsistent (Duque & Vázquez, 2015; Leyman et al., 2007), as demonstrated in Bodenschatz et al.’s (2021) study. Theoretically, given that depression is primarily characterized by low mood, the attentional focus in depression may be more specifically directed toward sad rather than angry faces (Duque & Vázquez, 2015; Sanchez et al., 2013; Suslow et al., 2020). Therefore, employing sad faces that more directly reflect core depressive symptomatology is necessary to further investigate attentional allocation patterns in demand-based visual search tasks, thereby testing the stability of task demand effects across different stimulus types. Furthermore, most existing eye-tracking studies utilize limited set sizes. Implementing varied set sizes not only enables examination of attentional allocation amidst multiple competing stimuli but also enhances the robustness and ecological validity of the findings (Basel et al., 2022; Suslow et al., 2020).

To achieve this, we consider how task demands influence processing modes. In passive viewing, spontaneous biases dominate, while in active visual search, top–down control can override these biases (Bodenschatz et al., 2021). However, perceptual load plays a crucial role in modulating cognitive resources available for control. A low load is too trivial to reveal individual differences because it relies on low-level feature detection, which reduces emotional salience (Nummenmaa & Calvo, 2015). Conversely, a high load depletes cognitive resources and hinders control, thereby masking emotional biases (Murphy & Zajonc, 1993). An intermediate load may create conditions where individual differences in regulatory capacity could emerge (Basel et al., 2022). Additionally, stimulus type affects the salience of emotional signals; this regulatory dynamic may be particularly relevant for sad faces, which serve as primary mood-congruent stimuli for depression (Duque & Vázquez, 2015).

Given the limitations of other experiments that have tested attentional bias in MDD, including the inconsistent use of sad faces, the narrow range of set sizes, and the insufficient separation of early orienting from late disengagement, we aimed to investigate these issues more systematically. Subclinical depression—characterized by elevated depressive symptoms without meeting full diagnostic criteria—is not merely a milder form of major depressive disorder (MDD) but may involve distinct cognitive processes. Individuals in this stage often maintain intact or even enhanced regulatory capacities (Dedovic et al., 2016), potentially allowing them to compensate for negative affect through adaptive attentional strategies. Understanding these processes in subclinical populations is critical for early intervention; however, few eye-tracking studies have specifically targeted this group with a focus on task demands and perceptual load. This study aims to address three key gaps: (1) whether the moderating effect of task demands observed with angry faces generalizes to sad faces, which are more relevant to depression; (2) whether attentional bias patterns remain consistent across different perceptual loads (set sizes of 4, 8, and 12), a factor not previously tested in subclinical depression; and (3) whether early orienting and late disengagement can be dissociated within a single paradigm to clarify the temporal dynamics of attentional bias. By addressing these gaps, we seek to provide a more nuanced understanding of attentional processes in subclinical depression.

The present study employed an eye-tracking face-in-the-crowd (FITC) task with participants varying in depressive symptoms to examine their attention allocation toward emotional faces. To systematically assess how task demands interact with stimulus characteristics and perceptual load, we introduced two key modifications to the Bodenschatz et al. (2021) paradigm. First, we substituted angry faces with sad faces. This allows us to discern whether divergent findings between visual search and free-viewing paradigms stem from the task demands themselves or from the specific emotional stimuli employed (Duque & Vázquez, 2015; Wisco et al., 2012). Second, to enhance ecological validity and test the generalizability of findings, we incorporated multiple set sizes (4, 8, and 12 faces). This manipulation examines the stability of attentional bias under varying levels of perceptual load (Basel et al., 2022). A consistent pattern of bias across different set sizes would strengthen the conclusion that task demands are the primary modulating factor. Finally, to precisely delineate the temporal dynamics of attentional bias—a point of contention in existing meta-analyses (Armstrong & Olatunji, 2012; Suslow et al., 2020)—the task was divided into two distinct phases. Part one required searching for an emotional target among neutral distractors to capture early attentional orienting. Part two required searching for a neutral target among emotional distractors to probe late-stage attentional disengagement.

As suggested by prior work, we hypothesized that, compared to the low depression group, individuals with high depressive symptoms would exhibit an attentional bias toward sad faces during the visual search task that, (a) in the early orienting phase (searching for emotional targets), facilitated the search for sad faces (shorter dwell time on distractors) but impaired the search for happy faces (longer dwell time on distractors); and (b) in the late disengagement phase (searching for neutral targets), would have difficulty disengaging from sad distractors (longer dwell time) but show active avoidance of happy distractors (shorter dwell time). This pattern would support the negativity-bias framework in depression (Duque & Vázquez, 2015). An alternative competing hypothesis is that explicit task demands engage top–down cognitive control, which may override any latent negativity bias in subclinical depression, leading to null group differences across all conditions (Bodenschatz et al., 2021).

## 2. Materials and Methods

### 2.1. Participants

The sample size was determined a priori using MorePower 6.0 (Campbell & Thompson, 2012). For a 2 (group) × 2 (face valence) × 3 (set size) mixed-design ANOVA, a total of 80 participants were required to achieve 80% statistical power (α = 0.05) for detecting a medium effect size ($\eta_{p}^{2}$ = 0.06).

Participants were recruited from a university student population via an online platform. They completed the Chinese version of the Patient Health Questionnaire-9 (PHQ-9; Min et al., 2013) along with a demographic information form on the Wenjuanxing platform (www.wjx.cn). Based on the methodology outlined by Basel et al. (2022) and related studies, we expanded our sample selection to include individuals with mild depressive symptoms, rather than limiting it to those with moderate or severe cases. This approach allows for a more comprehensive comparison of attention bias patterns in individuals at the subclinical stage, using a PHQ-9 score of 5 as the critical threshold. This cutoff aligns with established thresholds demonstrating high sensitivity and specificity (Kroenke et al., 2001; Ghazisaeedi et al., 2022). By including individuals with mild symptoms (PHQ-9 = 5–9), who are often excluded in studies that use higher thresholds (e.g., ≥10), we provide a more complete characterization of attentional patterns across the subclinical spectrum.

Participants scoring above 5 were assigned to the high depressive symptom (HD; subclinical range) group, while those scoring below 5 were assigned to the low depressive symptom (LD) group. Before the experiment, exclusion criteria were clearly communicated to participants to ensure the validity of the results. Exclusion criteria also included: (a) current or past diagnosis of any major psychiatric disorder (other than depression, assessed by self-report), (b) current use of psychoactive medications (e.g., antidepressants, anxiolytics), (c) history of neurological conditions or head trauma, and (d) failure to complete the eye-tracking calibration successfully. Thus, while participants in the HD group reported elevated depressive symptoms on the PHQ-9 (≥5), none had a formal diagnosis of MDD or were receiving psychiatric treatment, consistent with the operational definition of subclinical depression. Current mood state was not experimentally manipulated; all participants were tested in a neutral laboratory setting without mood induction.

Eighty participants were initially recruited. Data from three participants were excluded due to poor eye-tracking data quality, resulting in a final sample of 77 participants. The HD group consisted of 32 participants (mean age = 22.09 ± 2.43 years; 10 males) and the LD group consisted of 45 participants (mean age = 22.67 ± 2.52; 21 males). All participants reported normal or corrected-to-normal vision, no color blindness, right-handedness, and no history of mental illness, and had never received a diagnosis of major depressive disorder. Informed consent was obtained from all participants prior to the experiment, and monetary compensation was provided upon completion. The study protocol was approved by the university’s institutional ethics committee.

### 2.2. Materials

#### 2.2.1. Depression Measure

Depressive symptoms were assessed using the Chinese version of the Patient Health Questionnaire-9 (PHQ-9; Min et al., 2013). This self-report instrument comprises 9 items, each rated on a scale from 0 (“not at all”) to 3 (“nearly every day”). The total score ranges from 0 to 27, with established cut-offs indicating no depression (0–4), mild (5–9), moderate (10–14), moderately severe (15–19), and severe depression (20–27). In the present study, the internal consistency was good, with a Cronbach’s α of 0.821.

#### 2.2.2. Stimuli for the Face-in-the-Crowd (FITC) Task

All facial stimuli were selected from the Chinese Facial Affective Picture System (CFAPS; Wang & Luo, 2005). The selection was based on normative ratings of emotional intensity (on a 9-point scale, where 1 represents the weakest and 9 the strongest intensity) and was gender-balanced.

Emotional Face Search Task: For the task requiring participants to search for a single emotional target (sad or happy) among neutral distractors, 24 sad faces (M = 5.764, SD = 0.720), 24 happy faces (M = 5.268, SD = 0.731), and 22 calm faces (M = 5.739, SD = 0.185) were selected for the main experiment. An additional 8 sad, 8 happy, and 12 neutral faces were chosen for the practice session.

Neutral Face Search Task: For the task requiring participants to search for a single neutral target among emotional distractors (sad or happy), a distinct set of 24 calm faces (M = 5.804, SD = 0.150), 22 sad faces (M = 5.377, SD = 0.823), and 22 happy faces (M = 5.471, SD = 0.601) were selected for the main experiment. An additional 12 sad, 12 happy, and 8 neutral faces were used for practice.

### 2.3. Experimental Procedure

#### 2.3.1. FITC Task: Searching for Emotional Faces

**Figure 1:**
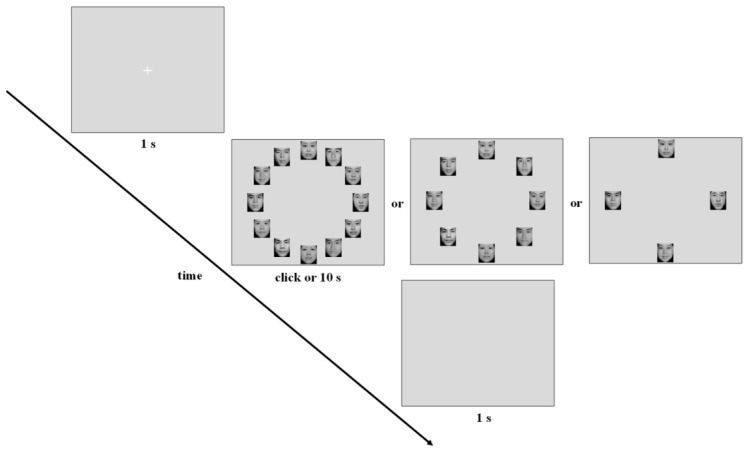
Experimental procedure flowchart for the emotional face search task. The example depicts a trial requiring participants to find a happy face among neutral distractors. The procedure for the neutral face search task was identical, except participants searched for a neutral face among emotional distractors.

Each trial in the emotional target search task began with a white fixation cross (2° × 2°) displayed for 1 s at the center of a gray screen. Subsequently, an array of 4, 8, or 12 face pictures (3.59° × 4.10°) was presented. Participants were instructed to identify the single face with a different emotional valence (either sad or happy) among neutral distractors and select it with a mouse click. A blank screen was displayed for 1 s following the response or after a 10 s timeout (Figure 1).

All faces were evenly distributed along an imaginary circle (radius: 450 pixels) centered on the screen, with 24 predefined positions separated by 15° increments. The angular separation between adjacent pictures was 30° for 12-face displays, 45° for 8-face displays, and 90° for 4-face displays. The number and position of faces were randomized across trials.

The session began with 48 practice trials, followed by 216 experimental trials. The experimental trials were equally distributed across the 2 (target valence: sad, happy) × 3 (set size: 4, 8, 12) conditions (36 trials per condition), organized into three blocks of 72 trials each, with a self-paused rest period between blocks to minimize fatigue. Trial order was fully randomized within each block to prevent order effects. Each emotional face was presented a maximum of twice, while the neutral distractor faces remained consistent across trials with randomized positions.

#### 2.3.2. FITC Task: Searching for Neutral Faces

The procedure for the neutral target search task was identical to the emotional target task, except for the roles of the stimuli. In this version, each trial contained a single calm/neutral face presented among emotional distractors (all sad or all happy faces). Participants were required to locate and select the neutral target.

The trial structure, spatial arrangement, and timing parameters were consistent with the emotional face search task. Participants completed 48 practice trials followed by 216 experimental trials, equally distributed across the 2 (distractor valence: sad, happy) × 3 (set size: 4, 8, 12) conditions, and divided into three blocks, with a self-paused rest period between blocks to minimize fatigue. Trial order was fully randomized within each block to prevent order effects.

#### 2.3.3. Apparatus

Eye movement data were recorded using a Tobii TX300 eye tracker (Tobii, Stockholm, Sweden). The experiment was programmed in PsychoPy3 (Version 2020.2.6) and presented on a 23-inch monitor with a resolution of 1920 × 1080 pixels and a refresh rate of 60 Hz. Participants were seated approximately 60 cm from the screen, and gaze data were collected at a sampling rate of 300 Hz.

### 2.4. Data Analysis

Behavioral trials with no response or incorrect responses, as well as extreme RTs more than four standard deviations from the mean, were excluded from analysis as outliers (Eastwood et al., 2001). In the FITC task: searching for emotional faces, the proportion of extreme values removed was 0.11%, while in the FITC task: searching for neutral faces, this proportion was 0.15%. Eye movement data were processed following the preprocessing pipeline established by Otero-Millan et al. (2014). Trials with less than two-thirds of valid gaze samples were excluded. This led to the removal of 3.62% of trials in the emotional-target search task and 4.34% of trials in the neutral-target search task. Areas of Interest (AOIs) were defined according to the presentation position in each trial, centered on each image location with a boundary matching the image dimensions (3.59° × 4.10°). The number of AOIs corresponded to the set size (e.g., 12 AOIs for 12-face displays). Only fixations falling within these picture-based AOIs were included in subsequent analyses. AOIs were classified as either target or distractor AOIs, and fixations outside all AOIs were excluded. A fixation was defined as maintaining gaze within a 1° visual angle for a minimum duration of 100 ms.

To better dissociate early orienting to emotional stimuli from late disengagement, we employed two variants of the FITC task. In the emotional-target search task, participants searched for an emotional face (sad or happy) among neutral distractors. This condition is thought to tap into attentional orienting toward emotional cues, given that emotional faces typically capture attention more readily than neutral faces. In the neutral-target search task, participants searched for a neutral face among emotional distractors (all sad or all happy). This condition is thought to tap into attentional disengagement from emotional stimuli, as emotional distractors are expected to produce greater interference and thus slower disengagement compared to neutral distractors.

Following the approach of Bodenschatz et al. (2021) and extending it with additional metrics, we selected the following eye-movement indices: (1) reaction time and (2) target fixation latency (time from stimulus onset to first fixation on the target) were used to assess search efficiency. In the emotional-target search task, shorter latencies indicate faster orienting to emotional targets. In the neutral-target search task, shorter latencies suggest more efficient disengagement from emotional distractors (i.e., less interference). (3) Average fixation duration on the target (in the emotional-target search task) and (4) on distractors (in the neutral-target search task) were examined to evaluate the attentional hold of emotional stimuli. In the emotional-target search task, longer average fixation on an emotional target may reflect stronger capture by that emotion. In the neutral-target search task, longer average fixation on emotional distractors may indicate difficulty disengaging from negative or positive information. (5) Total dwell time on the target was analyzed as an index of overall processing time required for target discrimination. Visual search involves not only initial orienting but also subsequent comparison and verification processes. In the emotional-target search task, shorter total dwell time on an emotional target may reflect more efficient identification and confirmation of that emotional face. In the neutral-target search task, shorter total dwell time on the neutral target (when emotional distractors are present) may indicate faster disengagement from emotional distractors and less interference from them.

We acknowledge that these eye-movement metrics are not process-pure; each may reflect a mixture of attentional processes (orienting, engagement, disengagement, and decision-making). Nevertheless, the task contrast (emotional-target vs. neutral-target search) provides a theoretically grounded framework within which these indices can be interpreted as broadly indicative of early orienting and late disengagement, respectively.

For both reaction time and eye movement data, we conducted repeated-measures ANOVAs with a 2 (group: HD vs. LD) × 2 (face valence: sad vs. happy) × 3 (set size: 4, 8, 12) factorial design. When the three-way interaction (group × face valence × set size) was not significant, we conducted exploratory analyses of the group × face valence interaction separately at each set size, based on theoretical interest in potential set-size-specific patterns. To address multiple comparisons, we applied the Benjamini–Hochberg false discovery rate (FDR) correction with q = 0.05 to all exploratory post hoc tests and correlational analyses (Benjamini & Hochberg, 1995). We visually inspected Q-Q plots for each condition and found no severe deviations from normality. Given the robustness of ANOVA, no transformations were applied. The Greenhouse–Geisser correction was applied when sphericity was violated, and Bonferroni correction was used for post hoc comparisons. Reported *p*-values are corrected accordingly. Analyses were performed separately for the emotional face search task (assessing early attentional bias) and the neutral face search task (assessing late attentional bias). All eye movement data processing was implemented using MATLAB (MathWorks, version R2020b).

## 3. Results

### 3.1. Demographic Information

As shown in Table 1, the two participant groups showed no significant differences in age or gender ratio. The only significant difference was in PHQ-9 scores, where the HD group scored higher than the LD group, *t*_(75)_ = 10.014, *p* < 0.001, Cohen’s *d* = 2.639.

### 3.2. FITC Task: Searching for Emotional Faces

**Figure 2:**
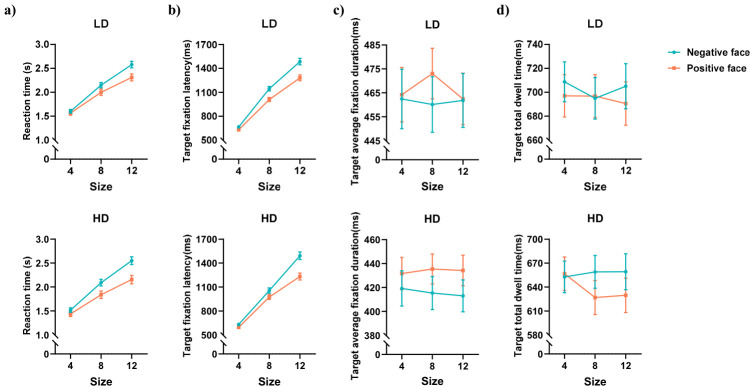
Descriptive statistical results for the emotional face search FITC task. (**a**) Reaction time; (**b**) target fixation latency; (**c**) target average fixation duration; (**d**) target total dwell time; LD = low depressive symptoms group, HD = high depressive symptoms group.

**Figure 3:**
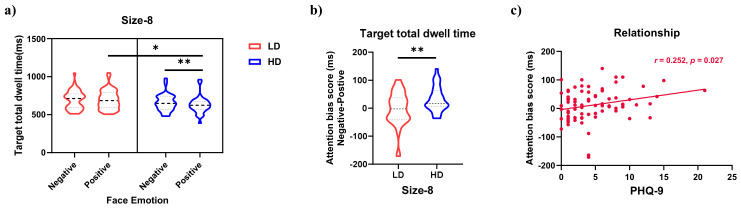
Interaction effect for target total dwell time and its correlation with PHQ-9 scores. (**a**) Group × face valence simple interaction in the 8-face condition of the emotional-target search task. Error bars represent standard errors; (**b**) attentional bias index (sad minus happy total dwell time) for the 8-face condition; higher values indicate shorter dwell time on happy faces (faster discrimination); (**c**) scatter plot showing the positive correlation between the bias index and PHQ-9 scores (*r* = 0.252, *p* = 0.027), indicating that higher depression severity was associated with faster discrimination of happy faces; LD = low depressive symptoms group, HD = high depressive symptoms group. (** *p* < 0.01, * *p* < 0.05).

The behavioral and eye movement results for the emotional face search FITC task are shown in Figure 2 and Figure 3.

#### 3.2.1. Reaction Time

As shown in Figure 2a, the main effect of face valence was significant, *F*_(1,75)_ = 44.543, *p* < 0.001, $\eta_{p}^{2}$ = 0.373. Reaction time was longer for sad faces than for happy faces (*p* < 0.001). The main effect of set size was significant, *F*_(2,150)_ = 732.966, *p* < 0.001, $\eta_{p}^{2}$ = 0.907. Reaction time for 12 faces was longer than for 8 and 4 faces (*ps* < 0.001), and reaction time for 8 faces was longer than for 4 faces (*p* < 0.001). The interaction between face valence and set size was significant, *F*_(2,150)_ = 40.432, *p* < 0.001, $\eta_{p}^{2}$ = 0.350. Follow-up analyses showed that reaction times for sad faces were consistently longer than for happy faces across all set sizes (*ps* < 0.024). The main effects and interactions involving other variables were not significant.

#### 3.2.2. Target Fixation Latency

As shown in Figure 2b, the main effect of face valence was significant, *F*_(1,75)_ = 120.962, *p* < 0.001, $\eta_{p}^{2}$ = 0.617. Target fixation latency was longer for sad faces than for happy faces (*p* < 0.001). The main effect of set size was significant, *F*_(2,150)_ = 1047.269, *p* < 0.001, $\eta_{p}^{2}$ = 0.933. Target fixation latency for 12 faces was longer than for 8 and 4 faces (*ps* < 0.001), and latency for 8 faces was longer than for 4 faces (*p* < 0.001). The interaction between face valence and set size was significant, *F*_(2,150)_ = 25.839, *p* < 0.001, $\eta_{p}^{2}$ = 0.256. Further analysis showed that target fixation latency was longer for sad faces than for happy faces across all set sizes (*ps* < 0.001). The main effects and interactions involving other variables were not significant.

#### 3.2.3. Target Average Fixation Duration

As shown in Figure 2c, the main effect of face valence was significant, *F*_(1,75)_ = 5.341, *p* = 0.024, $\eta_{p}^{2}$ = 0.066. The target average fixation duration was shorter for sad faces than for happy faces (*p* = 0.024). The main effects and interactions involving other variables were not significant.

#### 3.2.4. Target Total Dwell Time

As shown in Figure 2d, the main effect of face valence was significant, *F*_(1,75)_ = 12.468, *p* < 0.001, $\eta_{p}^{2}$ = 0.143. Target total dwell time was longer for sad faces than for happy faces (*p* < 0.001). The three-way interaction between group, face valence, and set size was significant, *F*_(2,150)_ = 3.601, *p* = 0.030, $\eta_{p}^{2}$ = 0.046. To decompose this interaction, we examined the group × face valence interaction at each level of set size. This analysis revealed a significant simple interaction effect only in the 8-face condition (Figure 3a), *F*_(1,75)_ = 7.531, *p* = 0.008, FDR-corrected *p* = 0.012, $\eta_{p}^{2}$ = 0.091. Between-group comparisons showed that for sad faces, there was no significant difference in target total dwell time between groups; for happy faces, the HD group had shorter target total dwell time than the LD group (*p* = 0.014). Within-group comparisons showed that the LD group had no significant difference in target total dwell time between face valences, whereas the HD group had shorter target total dwell time for happy faces compared to sad faces (*p* = 0.001). The group × face valence interaction was not significant in the 4-face and 12-face conditions. The main effects and other interactions were not significant.

The ANOVA results indicated that the HD group had shorter total dwell time on happy faces than the LD group. To further analyze between-group differences, an attentional bias index for target total dwell time was calculated for the 8-face emotional search condition. This bias index follows the difference-score method validated in previous visual search studies (Van Bockstaele et al., 2021), which has been shown to provide more reliable individual-difference estimates than traditional probe tasks. To quantify bias towards happy faces, the total dwell time on happy targets was subtracted from the total dwell time on sad targets. A higher index value indicates relatively shorter dwell time on happy faces, suggesting faster discrimination. An independent samples t-test on this attentional bias index (Figure 3b) revealed that the HD group had a larger bias index than the LD group, *t*_(75)_ = 2.744, *p* = 0.008, FDR-corrected *p* = 0.012, Cohen’s *d* = 0.635. Additionally, a supplementary correlation analysis between the bias index and PHQ-9 scores (Figure 3c) showed a significant positive correlation (*r* = 0.252, *p* = 0.027, FDR-corrected *p* = 0.027), indicating that higher depression levels were associated with faster discrimination of happy faces.

### 3.3. FITC Task: Searching for Neutral Faces

**Figure 4:**
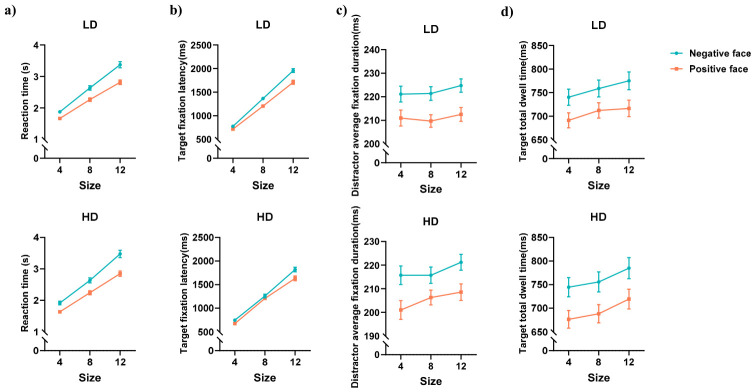
Descriptive statistical results for the neutral face search FITC task. (**a**) Reaction time; (**b**) target fixation latency; (**c**) distractor average fixation duration; (**d**) target total dwell time; LD = low depressive symptoms group, HD = high depressive symptoms group.

**Figure 5:**
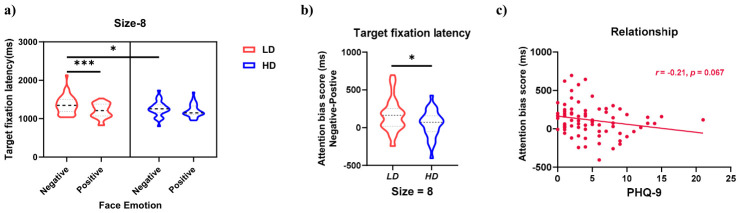
Interaction effect for target fixation latency and its correlation with PHQ-9 scores. (**a**) Group × distractor valence simple interaction in the 8-face condition of the neutral-target search task. Error bars represent standard errors; (**b**) attentional bias index (target latency with sad minus happy distractors) for the 8-face condition; higher values indicate greater interference (slower disengagement) from sad distractors; (**c**) scatter plot showing a marginally significant negative correlation between the bias index and PHQ-9 scores (*r* = –0.210, *p* = 0.067). This trend suggests that higher depression severity may be associated with faster disengagement from sad distractors, but the effect is weak and should be interpreted cautiously; LD = low depressive symptoms group, HD = high depressive symptoms group. (*** *p* < 0.001, * *p* < 0.05).

The behavioral and eye movement results for the neutral face search FITC task are shown in Figure 4 and Figure 5.

#### 3.3.1. Reaction Time

As shown in Figure 4a, the main effect of face valence (of distractors) was significant, *F*_(1,75)_ = 193.918, *p* < 0.001, $\eta_{p}^{2}$ = 0.721. Reaction time was longer with sad distractors than with happy distractors (*p* < 0.001). The main effect of set size was significant, *F*_(2,150)_ = 1189.075, *p* < 0.001, $\eta_{p}^{2}$ = 0.941. Reaction time for 12 faces was longer than for 8 and 4 faces (*ps* < 0.001), and reaction time for 8 faces was longer than for 4 faces (*p* < 0.001). The interaction between face valence and set size was significant, *F*_(2,150)_ = 28.427, *p* < 0.001, $\eta_{p}^{2}$ = 0.275. Further analysis showed that reaction time was longer with sad distractors than with happy distractors across all set sizes (*ps* < 0.001). The main effects and interactions involving other variables were not significant.

#### 3.3.2. Target Fixation Latency

As shown in Figure 4b, the main effect of face valence (of distractors) was significant, *F*_(1,75)_ = 65.261, *p* < 0.001, $\eta_{p}^{2}$ = 0.465. Target fixation latency was longer with sad distractors than *with* happy distractors (*p* < 0.001). The main effect of set size was significant, *F*_(2,150)_ = 1584.149, *p* < 0.001, $\eta_{p}^{2}$ = 0.955. Target fixation latency for 12 faces was longer than for 8 and 4 faces (*ps* < 0.001), and latency for 8 faces was longer than for 4 faces (*p* < 0.001). The interaction between face valence and set size was significant, *F*_(2,150)_ = 10.494, *p* < 0.001, $\eta_{p}^{2}$ = 0.123. Further analysis showed that target fixation latency was longer with sad distractors than with happy distractors across all set sizes (*ps* < 0.001).

Although the three-way interaction (group × face valence × set size) was not significant, analysis of the simple interaction effects between group and face valence at different set sizes revealed a significant interaction in the 8-face condition (Figure 5a), *F*_(1,75)_ = 5.719, *p* = 0.019, FDR-corrected *p* = 0.029, $\eta_{p}^{2}$ = 0.071. However, because this result stems from a post hoc decomposition of a non-significant three-way interaction, it should be interpreted with caution and considered preliminary. Between-group comparisons showed that with sad distractors, the HD group had shorter target fixation latency than the LD group (*p* = 0.034); with happy distractors, there was no significant difference between groups. Within-group comparisons showed that the LD group had longer target fixation latency with sad distractors than with happy distractors (*p* < 0.001), but the HD group showed no significant difference in target latency between the two distractor types. The group × face valence interaction was not significant in the 4-face and 12-face conditions.

The ANOVA results indicated that the HD group had shorter target fixation latency with sad distractors than the LD group. To further analyze between-group differences, an attentional bias index for target fixation latency was calculated for the 8-face neutral search condition. The calculation of this bias index followed the same difference-score approach as described for the emotional search task (Van Bockstaele et al., 2021). To quantify bias related to sad distractors, the target latency with happy distractors was subtracted from the target latency with sad distractors. A higher value indicates greater interference from sad distractors, i.e., slower disengagement. An independent samples t-test on this attentional bias index (Figure 5b) revealed that the LD group had a larger bias index than the HD group, *t*_(75)_ = 2.391, *p* = 0.019, FDR-corrected *p* = 0.029, Cohen’s *d* = 0.553. Additionally, a supplementary correlation analysis between the bias index and PHQ-9 scores (Figure 5c) showed a marginally significant negative correlation (*r* = −0.210, *p* = 0.067, FDR-corrected *p* = 0.067). Although not statistically significant, the trend indicated that higher levels of depression may be associated with faster disengagement from sad distractors. This result should be interpreted with caution due to its marginal significance and the exploratory nature of the analysis.

#### 3.3.3. Distractor Average Fixation Duration

As shown in Figure 4c, the main effect of face valence (of distractors) was significant, *F*_(1,75)_ = 145.415, *p* < 0.001, $\eta_{p}^{2}$ = 0.660. The average fixation duration on sad distractors was longer than on happy distractors (*p* < 0.001). The main effect of set size was significant, *F*_(2,150)_ = 6.079, *p* = 0.006, $\eta_{p}^{2}$ = 0.075. The average fixation duration on distractors for 12 faces was longer than for 8 faces (*p* = 0.002) and 4 faces (*p* = 0.015), while the difference between 8 and 4 faces was not significant. The main effects and interactions involving other variables were not significant.

#### 3.3.4. Target Total Dwell Time

As shown in Figure 4d, the main effect of face valence (of distractors) was significant, *F*_(1,75)_ = 99.482, *p* < 0.001, $\eta_{p}^{2}$ = 0.570. Target total dwell time was longer with sad distractors than with happy distractors (*p* < 0.001). The main effect of set size was significant, *F*_(2,150)_ = 14.499, *p* < 0.001, $\eta_{p}^{2}$ = 0.162. Target total dwell time for 12 faces was longer than for 8 faces (*p* = 0.003) and 4 faces (*p* < 0.001), while the difference between 8 and 4 faces was not significant. The main effects and interactions involving other variables were not significant.

### 3.4. Continuous Analysis with PHQ-9 Scores

To complement the group-based analyses and to preserve the full range of individual differences in depressive symptoms, we conducted linear regression analyses with PHQ-9 scores as the dependent variable. Based on the 8-face condition findings from the primary ANOVAs, we first examined the two raw eye-tracking measures that showed group differences: (1) target total dwell time on happy faces in the emotional-target search task, and (2) target fixation latency with sad distractors in the neutral-target search task. When entered separately as predictors, neither raw measure significantly predicted PHQ-9 scores (*ps* > 0.05), indicating that single-valence indices alone do not capture the relationship with depressive symptoms.

We therefore computed two attentional bias indices that contrast the two valence conditions: (1) for the emotional-target search task, the bias index = sad minus happy total dwell time (higher values indicate relatively shorter dwell time on happy faces); (2) for the neutral-target search task, the bias index = target latency with sad minus happy distractors (higher values indicate greater interference from sad distractors).

For the emotional-target search task, the bias index significantly predicted PHQ-9 scores, *b* = 0.019, 95% CI [0.002, 0.035], *β* = 0.252, *t*_(75)_ = 2.26, *p* = 0.027. The overall regression model was significant, *F*_(1,75)_ = 5.086, *p* = 0.027, adjusted *R*^2^ = 0.051. This indicates that a larger bias (i.e., relatively shorter dwell time on happy faces) is associated with higher depressive symptom levels.

For the neutral-target search task, the bias index showed a marginally significant negative relationship with PHQ-9 scores, *b* = −0.004, 95% CI [−0.009, 0.001], *β* = −0.210, *t*_(75)_ = –1.859, *p* = 0.067. The regression model was marginally significant, *F*_(1,75)_ = 3.456, *p* = 0.067, adjusted *R*^2^ = 0.031. This suggests a trend that faster disengagement from sad distractors (i.e., a smaller bias index) tends to accompany higher depression severity.

Notably, although the raw single-valence measures were not significantly associated with PHQ-9 scores, the valence-contrasting bias indices showed significant or marginal effects. One possibility raised by these findings is that the relationship between depressive symptoms and attentional processing might be more comprehensively captured by considering attentional biases that contrast different emotional valences (relative to neutral baselines), rather than focusing on any single emotional valence in isolation. Clearly, further research is needed to evaluate this interpretation. Together, these continuous analyses provide additional context to the group comparisons and indicate that the 8-face findings cannot be fully explained by the dichotomization of PHQ-9 scores alone, although replication is needed.

## 4. Discussion

The results revealed distinct attentional patterns between groups. In the emotional face search task (with neutral distractors), both the HD and LD groups demonstrated faster initial orientation toward happy faces across all set sizes. Critically, in the 8-face condition, the HD group showed a significantly shorter total dwell time on happy targets compared to the LD group. This pattern was corroborated by a significant positive correlation between the attentional bias index (sad minus happy total dwell time) and PHQ-9 scores, indicating that higher levels of depression were associated with facilitated identification of happy faces. In the neutral face search task (with emotional distractors), both groups exhibited greater attentional engagement with sad distractors, regardless of set size. Furthermore, in the 8-face condition, the HD group demonstrated shorter target fixation latency when searching among sad distractors compared to the LD group. A significant negative correlation emerged between the attentional bias index (target latency with sad minus happy distractors) and PHQ-9 scores, suggesting that higher depression levels were linked to faster disengagement from sad distractors during target search. It is important to note, however, that all significant group differences were restricted to the 8-face condition. In the emotional-target search task, no group effects were observed at set sizes 4 or 12; in the neutral-target search task, the three-way interaction (group × face valence × set size) was not significant, and the simple interaction at set size 8 was explored post hoc. Therefore, the interpretation of these findings as evidence for an adaptive regulatory mechanism should be considered preliminary and hypothesis-generating.

### 4.1. Adaptive Bias Towards Happy Faces in Early Orienting

Contrary to our hypothesis that individuals with high depressive symptoms would show facilitated search for sad faces and impaired search for happy faces, this study found no evidence of accelerated early orienting toward sad faces or attenuated orienting toward happy faces among individuals with high depressive symptoms during initial attentional engagement—a finding consistent with several previous reports (De Raedt & Koster, 2010; Gotlib & Joormann, 2010; Suslow et al., 2020). As much of the literature suggests, attentional bias in depression may manifest less in rapid detection of negative stimuli, and more in prolonged attentional maintenance once such stimuli are detected (Sanchez et al., 2013; Suslow et al., 2020). Such sustained attention could foster repetitive processing of negative information, potentially contributing to ruminative thought patterns that maintain depressive symptomatology (Donaldson et al., 2007; Duque & Vázquez, 2015). At the same time, these results may also reflect the moderating role of task demands. Under explicit task instructions, as in the present visual search paradigm, individuals with depression appear capable of performing search tasks similarly to non-depressed controls (Bodenschatz et al., 2021), which underscores the importance of considering how task demands themselves shape the expression of attentional bias. Based on the current results, the influence of task demands on early orienting appears robust, showing little modulation by variations in perceptual load or stimulus type.

Notably, in the 8-face condition, the high depression (HD) group demonstrated significantly shorter total dwell time on happy targets compared to the low depression (LD) group. One plausible interpretation is that this reflects reduced attentional engagement with positive stimuli in depression, aligning with existing evidence of diminished processing of positive information in affected individuals (Armstrong & Olatunji, 2012; Duque & Vázquez, 2015). Such attenuated engagement could impair the effective utilization of positive emotional cues for mood regulation, potentially exacerbating depressive symptoms (Moretta et al., 2021). However, the absence of between-group differences in average fixation duration on happy targets complicates this interpretation, suggesting that once attention is allocated to happy faces, the initial engagement may be comparable across groups. This pattern raises an alternative account related to task characteristics. In visual search paradigms, efficiency depends not only on initial orienting but also on subsequent comparison and confirmation processes (Wolfe & Horowitz, 2017). Thus, the shorter total dwell time observed in the HD group might indicate more efficient identification and discrimination of happy faces—a possibility that could reflect an adaptive regulatory mechanism in subclinical populations, wherein enhanced processing of positive information serves to mitigate negative affect (Dedovic et al., 2016).

Specifically, the attenuation of the positive attentional bias across the depression spectrum may not follow a linear trajectory; individuals at the subclinical stage may exhibit unique adaptive patterns (Dedovic et al., 2016). For instance, one study found that following a negative mood induction, individuals with a maternal history of depression showed increased attention toward negative faces, whereas those without such a history shifted their attention toward positive faces (Joormann et al., 2007). This suggests a protective mechanism, wherein individuals at lower risk for depression may adaptively bias their attention toward positive information to regulate negative mood. This distinction highlights a critical difference in attentional patterns between clinically diagnosed patients and non-clinical individuals with high depressive symptoms. The latter group, when faced with the explicit demands of a visual search task, demonstrated an attentional orienting advantage for positive information, potentially reflecting an enhanced capacity for adaptive regulation to mitigate negative emotion (Dedovic et al., 2016; Joormann et al., 2007). Furthermore, this aligns with evidence showing that attention bias modification training targeting positive information can effectively alter maladaptive attentional patterns in depression (Woolridge et al., 2021), underscoring the plasticity and clinical relevance of these mechanisms. Nevertheless, the current findings regarding happy-face processing were observed only in the 8-face condition; their reliability and generalizability await independent replication.

### 4.2. Attentional Avoidance of Sad Faces in Late Disengagement

Consistent with the findings of Bodenschatz et al. (2021), the present study did not observe increased attentional maintenance on sad faces during the late disengagement stage among individuals with high depressive symptoms. This supports the view that in goal-directed visual search paradigms, individuals with depression can perform comparably to non-depressed controls without exhibiting excessive sustained attention to negative stimuli (De Raedt & Koster, 2010; Gotlib & Joormann, 2010; Suslow et al., 2020). One potential explanation is that attentional bias toward negative information in depression may be less consistently observed than previously assumed. Research indicates that while anxiety is associated with rapid detection of threat, depression may be more characterized by memory biases for negative information (Marchetti et al., 2018). Alternatively, these divergent findings may reflect fundamental differences in experimental paradigms (Wisco et al., 2012; Lazarov et al., 2018). Unlike free-viewing tasks, visual search paradigms require active, goal-directed attention allocation, which may engage top–down cognitive control mechanisms that can override latent negative biases (Wisco et al., 2012). Crucially, our results demonstrate that this moderating effect of task demands on negative attentional bias remains consistent across different stimulus categories (sad vs. angry faces) and varying perceptual loads.

However, an unexpected finding emerged regarding target fixation latency: in the 8-face condition, the HD group showed a pattern of shorter target fixation latency, which may tentatively suggest facilitated disengagement from sad distractors, although this finding is exploratory and should not be overinterpreted. This result diverges from the pattern reported by Bodenschatz et al. (2021), a discrepancy that may be attributable to the subclinical nature of our sample, as attentional patterns in non-clinical populations often differ from those in diagnosed MDD patients (Dedovic et al., 2016). Notably, healthy individuals typically exhibit a protective attentional bias characterized by avoidance of excessive processing of negative information (Klawohn et al., 2020). Consequently, at the subclinical stage, individuals may employ compensatory strategies to alleviate negative affect by enhancing engagement with positive stimuli (Dedovic et al., 2016; Joormann et al., 2007). This interpretation aligns with spontaneous emotion regulation theory, which posits that individuals experiencing negative mood states may selectively attend to positive information as an adaptive coping mechanism (Forgas & Ciarrochi, 2002). Thus, in the early stages of depression, emotion regulation may operate through such adaptive attentional adjustments rather than through the maladaptive sustained attention to negative stimuli typically observed in clinical populations.

Given the high relapse rates in major depressive disorder and the limited efficacy of current cognitive bias modification interventions (Fodor et al., 2020), the potential for individuals in early stages of depression to employ adaptive regulatory strategies—enhancing positive information processing while reducing engagement with negative stimuli—highlights the crucial importance of early identification and intervention (Cuijpers et al., 2004). However, as suggested by Joormann et al. (2007), underlying risk factors such as family history may significantly influence an individual’s capacity to utilize positive information for mood regulation. This underscores the need to further explore additional moderating factors in attentional bias manifestation. The observed advantage in positive information processing among our participants may be attributable to their relatively mild depressive symptoms (mean PHQ-9 = 8.66), suggesting that such adaptive attentional patterns might be most evident in subclinical populations. Consequently, future research should employ larger sample sizes and conduct more refined analyses examining how attentional bias patterns vary across different levels of depressive severity.

The finding of facilitated disengagement from sad faces was limited to the 8-face condition and emerged only in exploratory analyses, given the non-significant three-way interaction; therefore, it should be interpreted with caution and requires replication before strong claims can be made. This study also found no significant group differences in attentional measures at the 4-face and 12-face conditions, and these null findings should not be considered evidence of an absence of attentional biases under varying perceptual loads. While our sample size (N = 77) was determined a priori to detect a medium effect size ($\eta_{p}^{2}$ = 0.06) with 80% power, it was insufficiently powered to detect small effects. Thus, the lack of significant differences at set sizes 4 and 12 may reflect this limitation rather than true equivalence. Future research with larger samples is necessary to determine whether attentional patterns differ across perceptual load levels in subclinical depression.

### 4.3. Adaptive Attentional Regulation in Subclinical Depression: Modulation by Task, Stimulus, and Load

The findings of this study demonstrate that attentional bias is subject to the influence of task demands (Wisco et al., 2012; Bodenschatz et al., 2021), as neither clinically depressed patients nor individuals with high depressive symptoms exhibited the expected enhanced negative bias or diminished positive bias in the visual search paradigm. Instead, the observed attentional patterns revealed a more complex picture: individuals with high depressive symptoms demonstrated accelerated discrimination of happy faces during early orienting alongside facilitated disengagement from sad faces in later processing stages. These results suggest that while task demands significantly shape the expression of attentional bias, the specific manifestations are further moderated by stimulus characteristics (Duque & Vázquez, 2015; Sanchez et al., 2013) and perceptual load conditions (Basel et al., 2022). It should be reiterated that the supportive evidence for this interpretation came solely from the 8-face condition.

The manifestation of negative attentional bias appears to be stimulus-specific. While individuals with high depressive symptoms in our study displayed rapid disengagement from sad faces during the late stage, this effect was not observed for angry faces in Bodenschatz et al. (2021), indicating that facilitated disengagement is not a universal response to all negative stimuli. Furthermore, the severity of depressive symptoms may be a critical moderating factor. The early processing advantage for happy faces could represent an adaptive regulatory mechanism in subclinical populations, where enhanced engagement with positive information serves to alleviate negative affect (Dedovic et al., 2016). This compensatory strategy may deteriorate as symptoms intensify, potentially explaining why such an advantage is not observed in clinically diagnosed MDD patients (Bodenschatz et al., 2021), a hypothesis that warrants further investigation.

Finally, the role of perceptual load cannot be overlooked. Both the facilitated processing of happy faces and the rapid disengagement from sad faces were specific to the 8-face condition. This suggests that set size is a key determinant in the expression of attentional bias. In the simpler 4-face condition, individuals may rely on low-level feature detection, diminishing the emotional salience of stimuli (Nummenmaa & Calvo, 2015). Conversely, in the more demanding 12-face condition, high perceptual load may consume cognitive resources, thereby masking emotional biases (Murphy & Zajonc, 1993). One speculative possibility is that the 8-face condition, representing an intermediate level of perceptual load, may be particularly sensitive to individual differences in emotional processing. However, this interpretation was neither pre-registered nor theoretically derived a priori; it emerged post hoc from the observed pattern of results. Because this effect was seen in only one set size, it should be treated with caution and tested directly in future studies that systematically manipulate perceptual load (e.g., by including a wider range of set sizes with explicit a priori predictions). We therefore do not place strong emphasis on this explanation, and the core finding remains that significant group differences were observed only in the 8-face condition—a pattern that itself requires independent replication.

In conclusion, attentional bias in depression is highly sensitive to experimental conditions, which contributes to inconsistent findings across studies. The expression of bias is mediated by task demands and further modulated by factors such as depression severity, stimulus type, and perceptual load. Crucially, the present findings suggest that during the early stages of depression, a potentially adaptive regulatory mechanism may be operative, whereby individuals compensate for negative affect by selectively enhancing the processing of positive information and facilitating disengagement from negative stimuli (Dedovic et al., 2016; Forgas & Ciarrochi, 2002). This protective orientation may help maintain psychological stability in subclinical populations, though it appears to diminish with the progression of the disorder.

### 4.4. Limitations

This study has several limitations that should be considered when interpreting the results. First, from the perspective of how task demands influence attention allocation in depression, our investigation was limited to a goal-directed visual search paradigm. Including a direct comparison with a free-viewing paradigm without explicit task demands would provide more compelling evidence for how attentional bias manifestations differ across varying requirement conditions (Bodenschatz et al., 2021; Lazarov et al., 2018). In addition, our interpretation regarding perceptual load is post hoc, and we report this pattern as an exploratory observation that awaits validation in larger samples with a pre-specified analytic plan. Future research should directly manipulate load levels to test whether the 8-face condition indeed yields the strongest emotional bias effects.

Statistical limitations also need to be acknowledged. While post hoc comparisons within each ANOVA were Bonferroni-corrected, no correction was applied across the entire set of tests (e.g., across tasks, simple-effect analyses, t-tests, and correlations). This is a limitation common to exploratory studies. Consequently, the significant effects restricted to the 8-face condition, particularly those from simple-effect analyses following non-significant three-way interactions, should be interpreted with caution. Independent replication with pre-registered hypotheses and appropriate control for multiple comparisons is needed to confirm these preliminary findings.

Furthermore, the absence of increased attentional engagement with negative information among individuals with high depressive symptoms in this study may be related not only to depression severity but also to current mood state. Some researchers propose that cognitive biases in depression may not be persistently active during routine information processing but might be specifically triggered under certain stressful conditions, potentially serving as a mechanism for inducing depressive symptoms (Segal & Ingram, 1994). Subsequent studies could incorporate negative mood induction procedures to further examine the impact of transient emotional states on attentional bias and validate the current findings (Clasen et al., 2013).

Finally, as the study population primarily consisted of non-clinical individuals with elevated depressive symptoms, the division into high and low symptom groups was necessarily based on a specific cut-off score—a methodology consistent with existing research (Basel et al., 2022). However, it is important to note that the high-symptom group encompassed a broad spectrum of severity, ranging from mild to severe depressive symptoms without formal diagnosis (Kroenke et al., 2001), and attentional bias patterns may vary considerably across this range. In addition, the sample was restricted to university students, which limits the generalizability of the findings to community or clinical populations. University students differ from community or clinical populations in terms of age range, cognitive functioning, socioeconomic status, and potentially the nature of depressive symptoms (e.g., academic-stress related). Therefore, the present findings may not generalize to older adults, clinical MDD patients, or individuals with lower educational attainment. Future research should employ finer gradations of depression severity and include more diverse samples, such as individuals with clinically diagnosed MDD and those from non-academic settings, to enhance the generalizability, precision, and replicability of these findings.

## 5. Conclusions

In summary, under explicit task demands, individuals with high depressive symptoms did not demonstrate the expected enhanced negative attentional bias. Paradoxically, they exhibited accelerated identification of happy faces during early orienting and facilitated disengagement from sad faces during late-stage processing. Specifically, individuals may maintain emotional stability through enhanced processing of positive information and attenuated engagement with negative stimuli. These effects were observed only in the 8-face condition and require replication. As such, these findings offer preliminary evidence that may be consistent with an adaptive cognitive regulatory mechanism in the early stages of depression development, but firm conclusions await independent validation. If replicated, these tentative patterns suggest that early-stage depression may involve adaptive attentional processes, which could eventually inform early identification efforts. However, no direct clinical implications can be drawn from the current data; future research is needed to determine whether these findings can be translated into intervention strategies.

## Figures and Tables

**Table 1:** Demographic Characteristics of the Two Groups.

	LD Group (*n* = 45)		HD Group (*n* = 32)	
	*M*	*SD*	*M*	*SD*
Gender(M:F)	21:24		10:22	
Age	22.67	2.52	22.09	2.43
PHQ-9	2.00	1.33	8.66	3.59

Note: LD = low depressive symptoms group, HD = high depressive symptoms group, PHQ-9, Patient Health Questionnaire-9.

## Data Availability

The original contributions presented in this study are included in the article. Further inquiries can be directed to the corresponding author.
